# Enhancing web search result clustering model based on multiview multirepresentation consensus cluster ensemble (mmcc) approach

**DOI:** 10.1371/journal.pone.0245264

**Published:** 2021-01-15

**Authors:** Ali Sabah, Sabrina Tiun, Nor Samsiah Sani, Masri Ayob, Adil Yaseen Taha

**Affiliations:** Center for Artificial Intelligence Technology (CAIT), Faculty of Information Science and Technology, Universiti Kebangsaan Malaysia, Bangi, Selangor, Malaysia; University of Engineering & Technology, Taxila, PAKISTAN

## Abstract

Existing text clustering methods utilize only one representation at a time (single view), whereas multiple views can represent documents. The multiview multirepresentation method enhances clustering quality. Moreover, existing clustering methods that utilize more than one representation at a time (multiview) use representation with the same nature. Hence, using multiple views that represent data in a different representation with clustering methods is reasonable to create a diverse set of candidate clustering solutions. On this basis, an effective dynamic clustering method must consider combining multiple views of data including semantic view, lexical view (word weighting), and topic view as well as the number of clusters. The main goal of this study is to develop a new method that can improve the performance of web search result clustering (WSRC). An enhanced multiview multirepresentation consensus clustering ensemble (MMCC) method is proposed to create a set of diverse candidate solutions and select a high-quality overlapping cluster. The overlapping clusters are obtained from the candidate solutions created by different clustering methods. The framework to develop the proposed MMCC includes numerous stages: (1) acquiring the standard datasets (MORESQUE and Open Directory Project-239), which are used to validate search result clustering algorithms, (2) preprocessing the dataset, (3) applying multiview multirepresentation clustering models, (4) using the radius-based cluster number estimation algorithm, and (5) employing the consensus clustering ensemble method. Results show an improvement in clustering methods when multiview multirepresentation is used. More importantly, the proposed MMCC model improves the overall performance of WSRC compared with all single-view clustering models.

## 1. Introduction

Information retrieval has become difficult due to the abundant online information currently available. Modern search engines respond to user queries with many results, and only a few prove to be relevant. Therefore, users are frequently compelled to browse through long lists of results. Web search engines perform excellently, but a typical user often ends up browsing numerous pages to find his needs. Research continues with numerous advances to make the process relatively fast and easy. Clustering strategies could be used by search engines to group the results of searches into various categories. In this manner, users may navigate directly to a category and find what they need faster than by browsing pages with a traditional web search engine. The abundance of the huge, continually increasing amount of information makes information retrieval an essential, difficult process. As the amount of available information increases daily, the amount of search results returned by a user query also increases. Nowadays, search result responds to user queries, of which only a few are relevant. Clustering is an effective technique to organize retrieval results in a meaningful cluster. However, existing clustering techniques have several drawbacks. Web search result clustering (WSRC) brings several challenges. Thus, various studies have proposed adopting clustering methods [1], and several clustering algorithms have been proposed. Based on the literature [1, 2] and the results obtained by [3], no single clustering algorithm is appropriate for all types of data. No clustering method is suitable for all tasks, probably due to the different dynamics and nature of web search result datasets. The different clustering results are likely due to non-normally distributed data [1, 4, 5]. Combined different clustering algorithms in which the final clustering is selected from the candidate clustering solution with the maximum similarity from all candidate clustering solutions. Therefore, clustering algorithm combinations, such as the single clustering algorithm, are inappropriate for all data types, and existing clustering methods utilize only one representation at a time (single view). By contrast, documents can be represented by multiple views.

In addition, existing clustering methods that utilize more than one representation at a time (multiview) use representations with the same nature. Hence, using multiple views that represent data in totally different representations with clustering methods and create a diverse set of candidate clustering solutions is reasonable. Moreover, the poor performance obtained by the existing research may be because various user queries can result in a different number of clusters in searching clustering results [1, 4].

On this basis, an effective, dynamic short-text clustering method must consider combining multiple views of data including semantic view, lexical view (word weighting), and topic view as well as the number of clusters. The key contributions of this study are as follows: First, an enhanced multiview multirepresentation clustering model that combines different views and representation methods is proposed. The different view and representation methods include n-gram weighting, word embedding Word2vec, and Dirichlet multinomial mixture (DMM) topic to generate different candidate clustering solutions. Second, an efficient radius-based cluster number estimation algorithm is proposed for the dynamic estimation of the number of clusters in WSRC and to overcome the limitations of k-based clustering, in which the number of clusters must be predefined. Third, a multiview multirepresentation consensus clustering ensemble method (MMCC), is proposed.

The proposed method can be adapted for short text clustering from online social media platforms, such as Twitter, to help extract knowledge. Moreover, the idea of the proposed method in this research can be applied to data with different views. The proposed method can also be applied for data that refer to different representations to achieve a better performance compared with single-view data representation. Thus, it can be implemented in real-time WSRC applications.

This study is organized as follows: Section 2 provides an overview of the relevant studies. Section 3 presents the proposed method of multiview multirepresentation approach with consensus clustering (CC) ensemble to create different candidate solutions. Sections 4, 5, and 6 presents the results and discusses the conducted experiments. Section 7 concludes the study.

## 2. Related studies

The multiview clustering approach has drawn considerable attention in recent years, and it aims to exploit consensus and complementary information through multiple views [6]. Clustering of search results has become one of the most important strategies for extracting knowledge online [7]. Clustering of search results that typically return snippets of web results in thousands is often impractical. The key is to identify optimal universal stopping criteria for every query. Several researchers have suggested clustering methods that can resolve the problems of conventional clustering strategies and handling WSRC difficulties. In this section, an overview of advanced studies related to WSRC is provided. The issue has been addressed through diverse strategies, but the median partition-based strategy remains the most well-known approach for ensemble clustering thus far [1, 8–10]. A median partition-based strategy involves a lone candidate clustering solution that features maximum similarity among all clustering solution candidates. This strategy is selected as the ultimate clustering solution for this research.

Abdulameer et al. [3] introduced a new framework that enhances the performance of WSRC following the new wiki-based K-nearest neighbor (KNN) representation technique. The approach also offers a new unsupervised distributed word representation model, where every word is characterized by a vector of its semantically linked referents, including schematic expansions of user queries and word snippets. Using the new wiki- and KNN-based representation technique is recommended for resolving short-text problems and improving the general performance of WSRC. Lastly, the results of the suggested model are reviewed and compared with those of baseline methods to confirm the importance of the proposed WSRC approach.

Feng et al. [11] developed a new text clustering ensemble algorithm derived from semantic sequence algorithmic methods proposed for this research. Text clustering results from the application of K-means and semantic sequence algorithms were generated and combined in accordance with overlap coefficient similarity principles to produce the coassociation matrix among semantic sequences. Lastly, the final clusters were acquired by merging documents that correspond to similar semantic sequences in this matrix.

Wang [12] introduced an enhanced non-negative matrix factorization (NMF) algorithmic method to combine multiple clusters. First, the K-means algorithm was performed to partition the adjacent matrix of the hypergraph and acquire the indicator matrix, which was then imparted to NMF as the initial factor matrix. Second, NMF was conducted to obtain the basis and coefficient matrices. Lastly, clustering results were acquired using the elements in the coefficient matrix.

Abu-Jamous et al. [13] developed a new clustering paradigm based on a new binarization of the consensus partition matrix method. The technique exploits the results of multiple clustering tests across the same dataset to generate a single fuzzy consensus partition. They suggested tunable methods that binarize this partition mirror biological reality, such that certain genes may be assigned to numerous clusters, whereas others may not be assigned at all. This technique can express the relative tightness of multiple clusters to generate tight or wide overlapping clusters as well as extract the unique genes bearing the profiles of these multiple clusters concurrently.

Gravitational ensemble clustering (GEC) denotes an ensemble clustering technique developed by Sadeghian and Nezamabadi [8]. This approach uses ensemble and gravitational clustering principles to obtain superior clustering results. Thus, the GEC model uses several runs of the K-means algorithm using different parametric initializations to generate the set.

In recent years, the multiview clustering approach brought about a vital expansion of ensemble clustering methods. Clustering algorithms can be applied to varying views of data to obtain dissimilar cluster labels for similar sets of objects using the multiview clustering method. These methods are then combined such that the final clustering results generated are superior to the individual clustering results for each data multiview approach. Multiview clustering can be used in various steps of the proposed clustering model. Hussain et al. [14] introduced the multiview clustering algorithmic method, where different ensemble methods are combined for a better effect. The approach computes various similarity matrices for individual datasets and then aggregates these matrices to construct a combined similarity matrix that is subsequently used to acquire the final clustering results.

Wahid et al. [1] developed a new approach for cluster ensemble, namely, the multiview multiobjective cluster ensemble derived from the multiview multiobjective evolutionary algorithm. This approach accords with the development of crossover methods that produce new clusters for the candidate clustering solutions, the development of mutational techniques for margin clusters and splitting, and the improvement of multiobjective fitness functions for resolving multiobjective optimization problems. The main limitation of this strategy is that its implementation requires predefined multiple views. It also requires the investigation of automatic detections of multiview results in documents.

Boongoen & Iam-On [15] reviewed cluster ensemble approaches while covering new extensions and applications for a range of ensemble-generating strategies with summarizations and representations of ensemble members and discussion of the issues of CC. Their survey also included differing extensions and applications of cluster ensemble methods that focus on numerous research problems and challenges. The outcome obtained from the literature review of web search results showed that clustering is the most effective approach and an issue that encourages new solutions to increase effectiveness and efficiency with massive, heterogeneous, and dynamic web pages. However, despite much research in the field of web search result, several open issues, such as the achievement of a better quality and an effort to deal with the drawbacks of each technique, need further investigation. At present, the majority of multiview clustering methods are derived from single-view concepts, where one measure of quality for partitioning is optimized explicitly or implicitly using various paradigms for single-view learning. Current clustering techniques use only single representations at any time (single views), whereas documents can be represented by multiple views. Moreover, multiple representations may bring about multiple cluster sets and provide multiple insights into the data.

Multiview clustering has been successfully applied to numerous applications including computer vision [16–18], natural language processing [19, 20], social media [21–23], bioinformatics, and health informatics [24–26]. Thus, an efficient, dynamic short-text clustering technique should consider the combination of multiple views of data because different views in parallel have been proven to improve clusters [27]. Thus, using multiple views for producing multiple candidates in a clustering collection technique is logical. Therefore, in this study, a method to enhance the clustering techniques with a new multiview, multirepresentation clustering framework that integrates n-gram weighting, DMM, and word embedding Word2vec to generate multiple candidate clustering solutions is proposed. The following section describes the framework for enhancing the WSRC model based on our proposed method, the MMCC.

## 3. Proposed multiview multirepresentation cluster ensemble model

**Fig 1:**
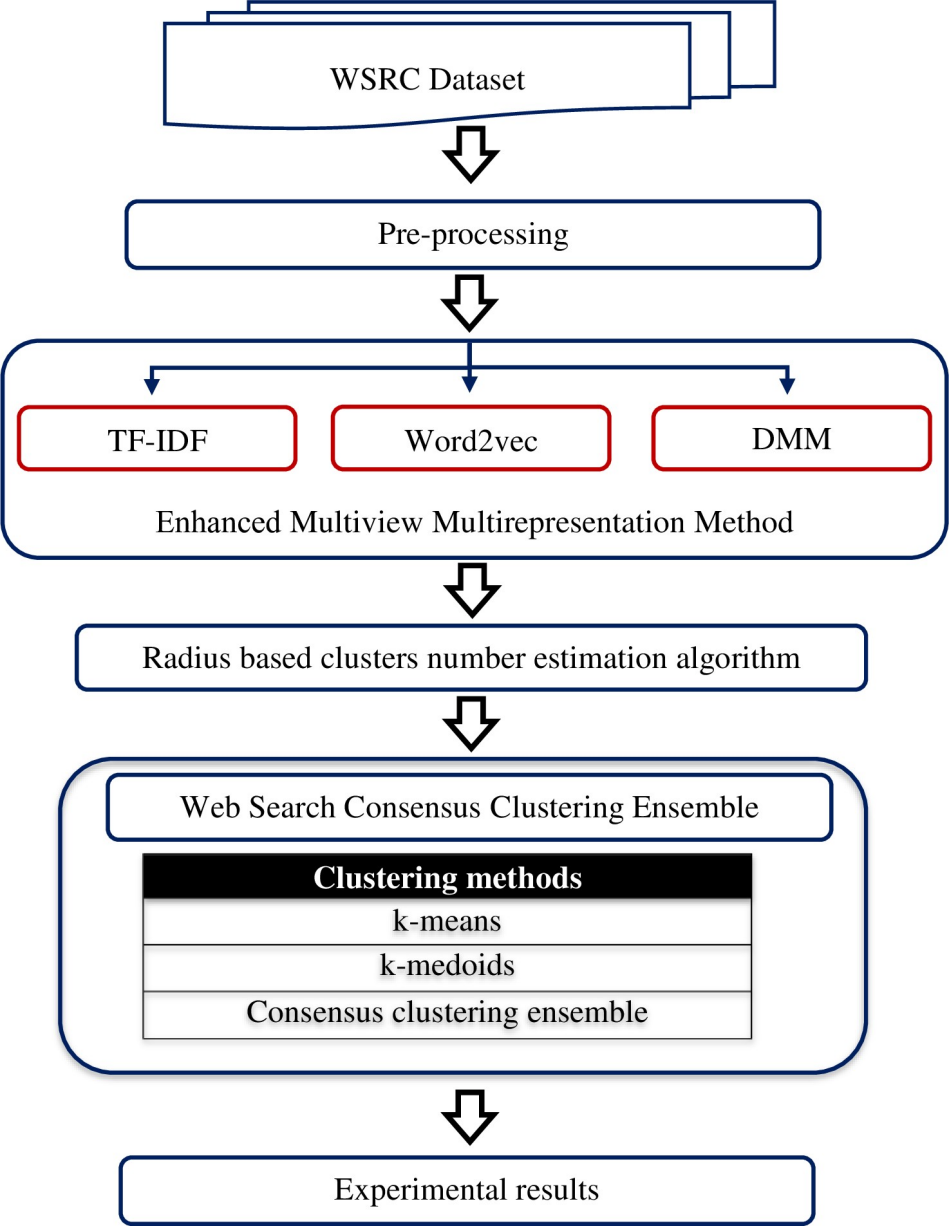
Proposed framework for enhanced WSRC model.

The section presents the detailed description of the framework for enhancing clustering models for WSRC based on the proposed multiview multirepresentation and the ensemble consensus clustering method (MMCC). The framework consists of six stages. Fig 1 describes the framework to develop the MMCC ensemble method and enhance the WSRC model.

The proposed framework includes several stages. The first stage is identifying and selecting appropriate datasets to be used. The datasets, Open Directory Project (ODP)-239 and MORESQUE, which are selected in our study to validate the clustering methods, were extensively used in several previous studies [28–32]. The second stage involves applying the preprocessing techniques for the datasets. The third stage is applying our proposed multiview multirepresentation clustering method, a combination of different views and representation methods (n-gram weighting, word embedding Word2vec, and DMM topic), to create different candidate clustering solutions. The fourth stage uses an efficient radius-based cluster number estimation algorithm to determine the dynamic number of clusters. The fifth stage proposes the MMCC method by combining different views and representation methods to generate various candidate clustering solutions. The last stage presents the standard evaluation metric to evaluate the proposed method.

### 3.1 Multiview multirepresentation for clustering models

Diverse representations result in different sets of clusters and may provide different data insights, considering that the multiview multirepresentation method was already proven to enhance clustering quality [27]. Recently, the trend moved toward formulating clustering ensembles as optimization problems to enhance the results [33, 34]. Hence, using multiple views that represent data in totally different representations with clustering methods and create a diverse set of candidate clustering solutions is reasonable.

**Fig 2:**
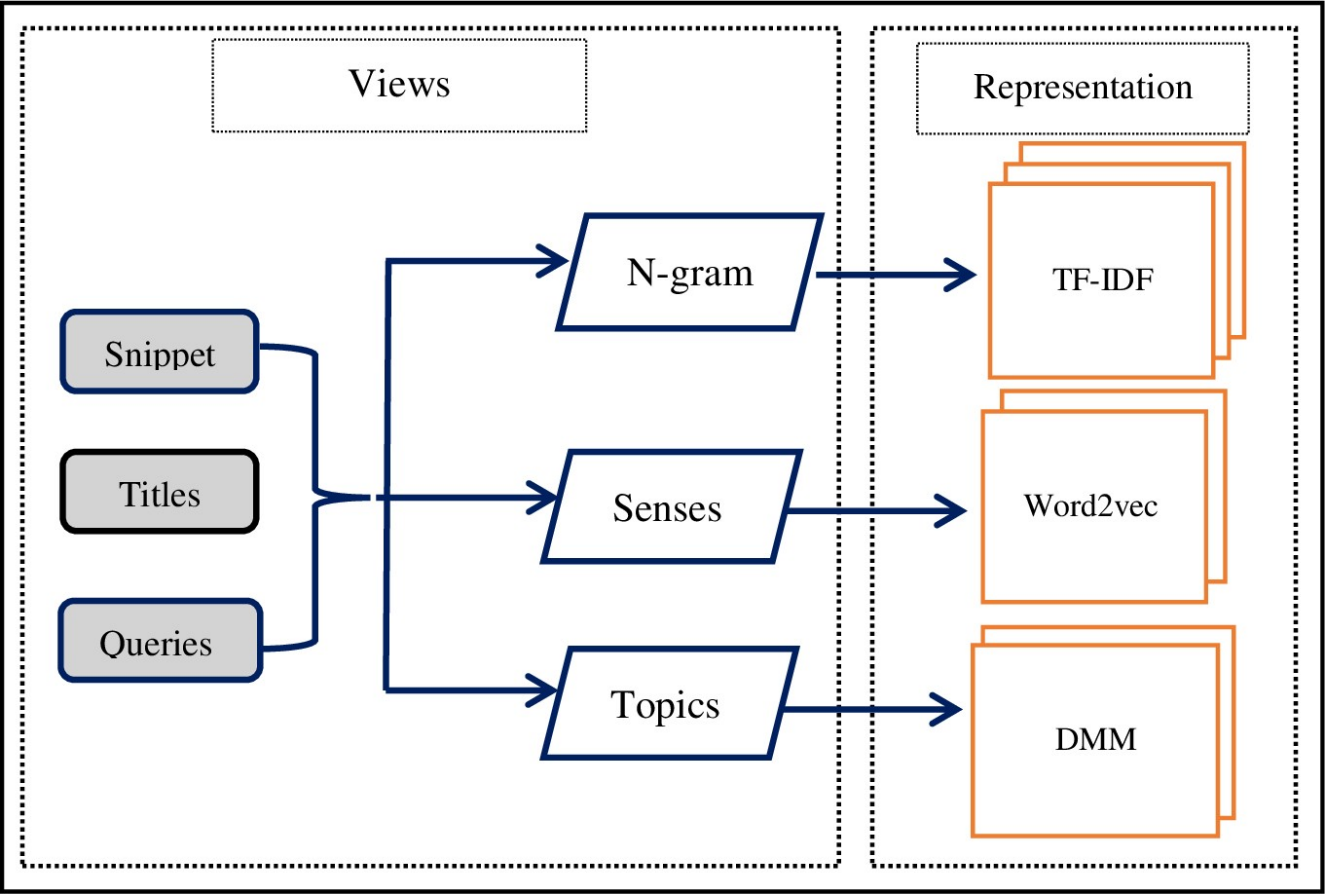
Proposed multiview multirepresentation models.

Multiview data refer to different representations of the same data instance. Each view may have diverse statistical properties that result in various insights of the data. Compared with previous clustering ensemble methods [1, 2, 35], this study adopts views and representations that are completely different to generate initial candidate clustering solutions. Thus, a multiview multirepresentation of data is introduced to create a clustering ensemble suitable for large data types. These representations and views ensure that different candidate clustering solutions are created by various clustering methods. In addition, cluster ensemble results are remarkably affected by the level of document representation [36]. Moreover, different representations may capture varying degrees of explanatory ingredients hidden in short documents. This study aims to enhance the performance of the clustering solution by selecting high-quality clusters from diverse candidate clustering solutions created by different clustering methods. From this perspective, several views of data are considered in the proposed approach, including n-grams, senses, topics of snippets, user query, and titles. In addition, three diverse representations, namely, term frequency–inverse document frequency (TF–IDF) matrix representation, word embedding representations (Word2vec), and latent topic representation (DMM), are used. The proposed multiview multirepresentation is shown in Fig 2.

These representation methods are selected due to the following reasons:

1. All representations have different statistical properties or formulations and come from diverse genres to ensure varied candidate clustering solutions.
2. Methods such as DMM derived from Latent Dirichlet Allocation are popular and still among the most effective representation methods [37, 38].
3. The most evident advantage of DMM and Word2vec representations is that they reduce the dimensions of features for representing a document [2, 37, 39].
4. Word2vec is a modern representation method that efficiently captures the implicit semantics of words [2, 39]. DMM also captures the implicit semantics of words [40].

In n-gram with TF–IDF representation matrix, every row represents a search result document, every column signifies an n-gram, and a cell contains the TF–IDF weighted value of an n-gram for a search result document. In word embedding representations, each search result document signifies a set of n vectors. Every vector represents a word from the search result document, and each vector of a word contains its most *k* similar words with their similarity values obtained using Word2vec. In a DMM, rows represent documents, and N columns denote N topics. These representations are obtained from different genres, and the n-gram with TF–IDF matrix representation is based on a simple numerical statistic. The word embedding representation is a word representation based on co-occurrence statistics and deep neural network. DMM is based on multinomial probability distribution theory.

#### 3.1.1 Query–snippet–title n-gram view and representation

In text clustering, a document is represented by a vector of terms and their weight values called attribute values. A common weighting scheme is assigned to each word based on TF–IDF [41–43]. A search engine generates a set of search results when using a query. Each search result consists of query, snippet, and title (QST). In query–snippet–title n-gram view and representation (QSTNVR), QST is combined and represents a bag of the n-gram. QST is tokenized into an n-gram list, which contains a list of unigram, bigram, and trigram. Each n-gram is weighted using the TF–IDF weighting scheme. A QST in n-gram is represented by Eq (1):

$$SR=[\begin{array}{c} TF-IDF(g_{1},SR)\\ TF-IDF(g_{2},SR)\\ TF-IDF(g_{3},SR)\\ \vdots \\ TF-IDF(g_{n},SR) \end{array}],$$ (1)

where *n* in *g*_*n*_ is the whole number of n-gram, and *TF*−*IDF* is the *TF*−*IDF* function defined by Eq (2):

$$TF-IDF(g_{i},SR)=tf(g_{i},SR)*\log\left(\frac{N}{df(g_{i})}\right),$$ (2)

where TF is the term frequency in document d, *IDF* is the number of appearances of this term in the document, *tf*(*g*_*i*_, *SR*) is the frequency of n-gram *g*_*i*_ in the search result, |N| is the whole number of search, and *df*(*g*_*i*_) is the number of search results with n-gram *g*_*i*_. QSTNVR representation uses the lexical representation of the search result document and neglects its semantic representations.

#### 3.1.2 QST Word2vec view and representation

Representations of word embeddings, such as Word2vec, are effective in describing fine-grained semantic associations between words [44]. Distributed word representation, Word2vec, interprets low-dimensional, closely packed word embeddings and efficiently captures the implicit semantics of words [2, 39, 40, 45, 46]. The low-dimensional embedding of words is much more appropriate for the latest deep learning neural-based models compared with conventional one-hot representation. Based on word embeddings, Word2vec represents every word denoted by ***x*** in a vocabulary, denoted by **V** as a low-dimensional compact vector, and denoted by **v**_**x**_ in space $R^{D}$. Such word representations can be learned based on the distributional concept, which assumes that words with related contexts are likely to have similar sense. Skip-gram’s training goal is to search for word representations, which are helpful in predicting the neighboring words in a statement from a large textual collection. Given a series of training words represented by {**ω**_**1**_, **ω**_**2**_,…,**ω**_**T**_}, the goal of the skip-gram model is to increase the mean log probability in Eq (3) [44, 47]:

$$J(\Omega )=\frac{1}{T}\sum_{t=1}^{T}\sum_{-c\leq j\leq c,j\neq 0}\log\;p(\omega_{t+j}|\omega_{t}),$$ (3)

where Ω represents the parameters of the model to be trained (defined later), *c* is the training context size (a sliding window surrounding the center word *ω*_*t*_), and *p*(*ω*_*t*+*j*_|*ω*_*t*_) denotes the probability of finding the word *ω*_*t*+*j*_ with respect to the center word *ω*_*t*_ and is defined by a basic hidden layer neural network model. The network has three strata, which include a hidden layer, an input layer, and a SoftMax output layer, corresponding to the words in the context window. Generally, the network serves as the input $\omega_{t}\in R^{V}$, where *V* represents the size of the vocabulary. It creates a hidden state represented by $h\in R^{D}$, where *D* denotes the hidden layer size or embedding space dimension, which is further converted to the output represented by $\omega_{t+j}\in R^{V}$. Multiple layers are completely connected, where the weight matrix *M*_*out*_ at the output strata is shared among all the contextual words. Gathering these weight matrices from this model represents the parameter of the model by $\Omega =\{M_{in}^{V\times D},M_{out}^{V\times D}\}.$ In the skip-gram architecture based on Eq (3), the input is represented by *ω*_*t*_ functions as a sparse vector of a one-hot (or 1-of-V) encoding where the element related to input *ω*_*t*_ is 1, and the remaining components are set to 0. Thus, the basic formulation of skip-gram defines *p*(*ω*_*t*+*j*_|*ω*_*t*_) as the function of SoftMax in Eq (4) [47]:

$$p(\omega_{O}|\omega_{I})=\frac{exp(\langle V_{in}^{\omega_{I}},V_{out}^{\omega_{O}}\rangle )}{\sum_{\omega =1}^{V}exp(\langle V_{in}^{\omega_{I}},V_{out}^{\omega_{O}}\rangle )},$$ (4)

where 〈.,.〉 represents the inner product amongst two vectors, $V_{in}^{\omega }$ and $V_{out}^{\omega }$, which are the “input” and “output” vectors of *ω*, respectively, and relate to the rows of the model parameter matrices denoted by *M*_*in*_ and *M*_*out*_.

#### 3.1.3 QSTDMM view and representation

**Fig 3:**
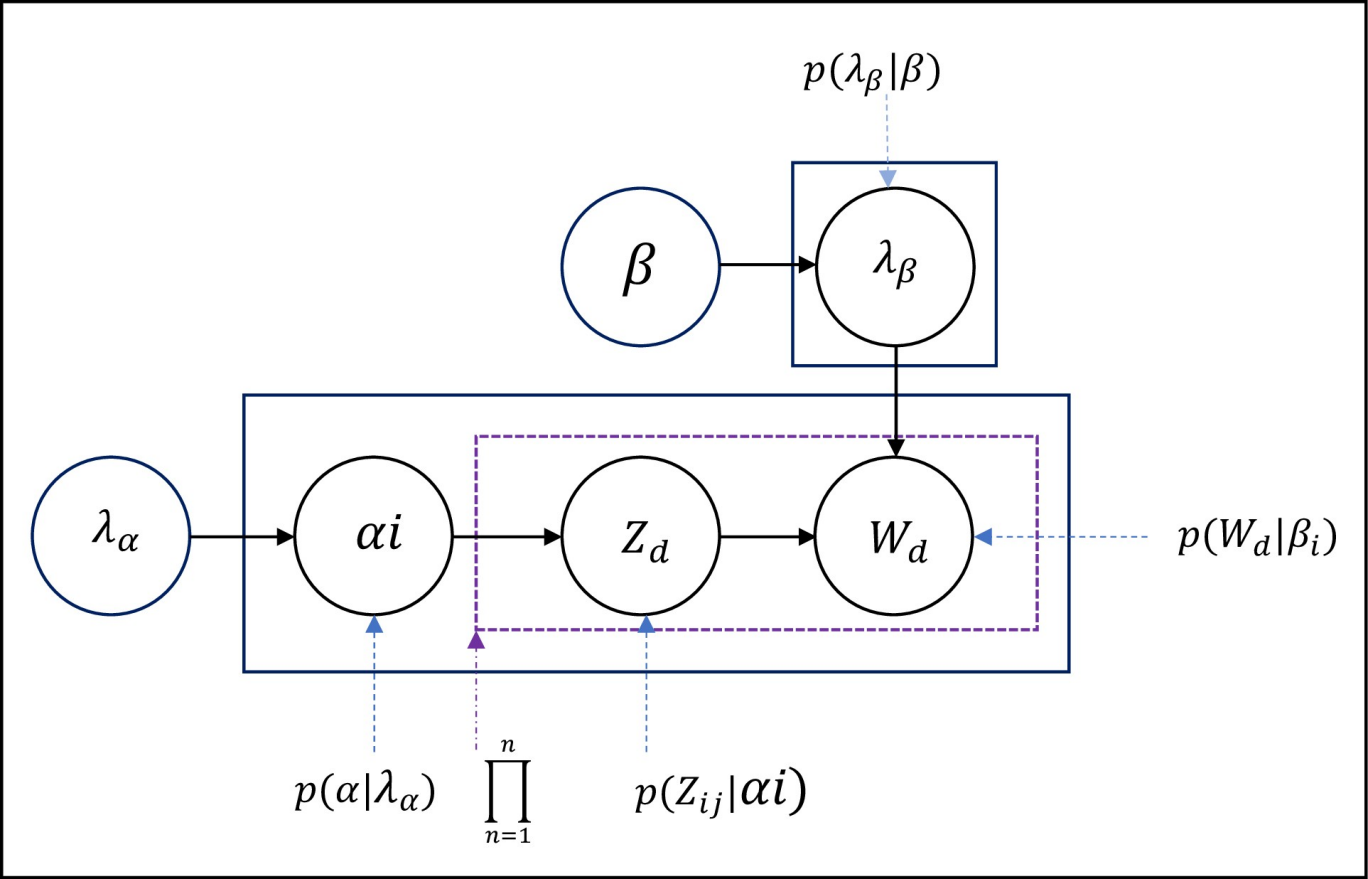
DMM models in the generative process.

The DMM model used in this study [37, 38, 48], a popular topic modeling method that is used to extract a topic from the documents, has semantic information. DMM typically refers to the mixture of the unigram model. In applying the DMM model for search result clustering purposes, QST is assumed to belong to one topic. Each snippet is associated with at most one topic. Corpus is a set of search results composed of ***D*** QSTs. ***D*** is the number of QST in the corpus. Each QST $\vec{d}$ contains a set of words (**wϵ1,2,..,N**_**d**_). The DMM model D in Fig 3 has the following generative process:

- *α* = (∝_1_, ∝_2_,…,∝_*n*_) is sampled from a Dirichlet distribution with parameter *λ* = (*λ*_∝1_, *λ*_∝2_,…,*λ*_∝*n*_).
- For each topic *t* = 1,2,…,*n*_*T*_, *β*_*t*_ is sampled from a Dirichlet (*λ*_*β*_), where *β* = (*λ*_*β*1_, *λ*_*β*2_,…,*λ*_*βn*_).
- For a QST $\vec{d}$ (*dϵ*{1,…,N}): in QST d,
  1. A topic *z*_*n*_∈{1,2,…,*T*} is selected from Multinomial(*α*) where *α* = (∝_1_, ∝_2_,…,∝_*n*_) represents the topic distribution in the corpus.
  2. Word count *N*_*d*_ is selected, and a word *w*_*d*_ from QST d from Multinomial(*β*) is independently selected, where *β* = (*β*_∝1_, *β*_∝2_,…,*β*_∝*n*_) represents the word topic distribution in the corpus.

The probability of observed data D is computed and maximized in Eq (5) to infer the latent variables and hyperparameters [16, 37]:

$$p(d|\alpha ,\beta )=\sum_{l=1}^{T}\alpha_{l}\frac{N_{d}!}{\prod_{w=1}^{V}N_{d}^{w}!}\prod_{w=1}^{V}\beta_{wt}^{N_{d}^{w}},$$ (5)

where *α* indicates the parameters of topic Dirichlet prior and the distribution of words over topics from the Dirichlet distribution given *β*, V is the size of the vocabulary, *N*_*d*_ is the number of words in the QST, and $N_{d}^{w}$ is the frequency of word w in QST *d*. The Dirichlet-multinomial pair for the corpus-level topic distributions is (*α*, *θ*).

In a QSTDMM output matrix, rows represent documents, and columns denote topics. A cell (i,j) is labelled 1 if document *d*_*i*_ is from topic *t*_*j*_. The most evident advantage of this representation is reducing the dimensions of features for representing a document.

### 3.2 Radius-based number of cluster estimation algorithm

WSRC is a dynamic clustering. Each query has its own search result documents. K-based clustering algorithms are more challenging in search result clustering, where every user’s queries can result in a different number of clusters. This study introduces a radius-based number of the cluster’s estimation algorithm to overcome the limitations of k-based clustering, where the number of clusters must be predefined. The stepwise flow of the proposed algorithm is summarized as follows (see Algorithm 1):

Step i: Inferring a search result graph: Given a query and its search result documents Q_*d*_ = {r_1_,….,r_m_}, each document is represented as a node. The edge between two nodes *e*_*ij*_ is straightforwardly computed between each pair of search result nodes (r_i_, r_j_) given a radius value *β*, which is a random number less than ≤0.5 using Eq (6) [49]:

$$we_{ij}=\left\{ \begin{array}{cc} \cos\left(r_{i},r_{j}\right)=\frac{\left|r_{i}|*|r_{j}\right|}{\sqrt{r_{i}^{2}*r_{j}^{2}}} & if\;\cos\left(r_{i},r_{j}\right)>\beta \\ 0 & otherwise \end{array}\right.$$ (6)

Step ii: Density and degree estimation: The density of each search result document *r*_*i*_ from the dataset is defined as the sum of the weight of all its edges and calculated using Eq (7) [49]:

$$Density(ri)=\sum_{k}\left(e_{ik}\right)$$ (7)

Step iii: Highest density document selection: The document or node ri, which has the highest density (Density(ri)>Density(rj); ∀j) is selected as the first seed S_j1_. All nodes connected to it are removed from the dataset and added to its cluster.

Step iv: Q optimal target cluster selection: Steps (iii) and (iv) are repeated to continue the selection of the subsequent highest density *document* as long as the graph still has nodes.

**Algorithm 1: Radius-based number of cluster estimation algorithm**

**Input:** Given a query and its search result documents Q_*d*_ = {r_1_,….,r_m_}

**Output: Set of candidate centroids C**

BEGIN

    ***Step i*: The graph similarity matrix WE is calculated**

    For each document pairs (*r*_*i*_, *r*_*j*_) in Q_*d*_

        *β*←*select_small_random_number*(0.50)

      *Begin*

            *sim*←*calclate_cosine_similarity*(*r*_*i*_, *r*_*j*_)

                *if*(*sim*>*β*)

        *WE*[*i*,*j*]←*sim*

                    *Else if*

        *WE*[*i*,*j*]←0

            *End if*

      *End*

    ***Step ii*: The document with the highest density is identified**

    *Density_max*←0

        *For i = 1 to n // n number of* search results documents

      *Begin*

            *Doc*_*density*←0

                *For j = 1 to n // n number of* search results documents

                    *Begin for*

                  *Doc*_*density*_←*Doc*_*density*_+*WE*[*i*,*j*]

                  *End for*

    ***Step iii*: The document with the highest density is selected**

      *if (Doc*_*density*_>*Density_max*)

            *Begin if*

      *Density_max*←*Doc*_*density*_

                *Dens*_*document*_←*ri*// remove all documents connected with *Dens*_*document*_

                                    *by making all of its row 0*

            *For k = 1 to n // n number of* search results documents

                        *If* (*WE*[*i*,*k*]>0)// if document *rk* is connected with document *ri*

                          *Begin if*

                              *For v = 1 to n // n number of* search result documents

                                    *WE*[*i*,*k*]←0

                            *End if*

                    *End if*

            *End*

    ***Step iv*: The document with the highest density is added to candidate centroids, and this process is repeated**

            ***Begin***

        *C*←{*Dens_document*}

        *repeat step* 2 *and* 3 until all entries in matrix WE are zeros

          *End*

     *End*

return C

### 3.3 Consensus clustering ensemble method

Cluster ensemble methods are learning techniques that create a cluster set to produce new information points by considering their predictions’ vote. A sufficient, necessary condition to obtain a more precise cluster collection than any individual member is that the clusters must be separate and accurate [50]. The chief finding is that such collections are often very precise than their individual clusters, and the combination of many single strategies improves the performance of the ultimate classifier reliability, accuracy, and understandability [51]. In addition to regression and categorization, ensemble techniques have been developed for clustering as well as other machine learning functions [9].

Collecting various clustering solutions is recommended, where diversity is produced by allowing every base clustering to be created using different clustering techniques. The main idea is that every clustering method has advantages and drawbacks, and is appropriate for certain types of datasets. Different clustering models can provide various decisions on clustering of data and support one another. Therefore, combining the outcomes of different clustering techniques can ensure better clustering of data. Multiple techniques can provide multiple decisions on data components and complement one another [1, 5, 34]. This study uses three views and representations, namely, QSTNVR, QSTWVR, and query–snippet–title DMM view and representation (QSTDVR) with k-based clustering methods, namely, k-means and k-medoids, to generate the ensemble. In addition, base clustering must be precise and varied to acquire a high-quality ensemble [9, 52, 53].

Cluster ensembles are effective for improving the robustness and stability of unsupervised clustering solutions [1, 34, 35, 54]. However, finding a CC to combine multiple-base clustering is the main objective of this study. In Algorithm 2, CC is applied on all solutions to generate a set of clustering solutions *p* = {*p*_1_, *p*_2_,….,*p*_*m*_}, where each clustering solution *p*_i_ = {*C*_1_,….,*C*_*K*_}. These different clustering solutions are created by k-means and k-medoids clustering algorithms with multiview and multirepresentation. QST, which agrees with *m* clustering solution, should be determined to achieve a consensus among P clustering methods (with clustering solutions). First, let *u* and *v* be two QSTs of *X*, *where X* = {*x*_1_, *x*_2_,⋯,*x*_*n*_} is a set of n QST. Then, the algorithm determines the frequency when QST *u* and *v* are clustered together in all clustering methods *P*. The algorithm determines the frequency when two objects are clustered given two clustering solutions p1 and p2 produced by two different clustering methods. Let *T* be the similarity measure, as follows [55]:

$$T_{u,v}(p_{i},p_{j})=\left\{ \begin{array}{cc} 1 & if\;p_{i}(u)=p_{i}(v)\;and\;p_{j}(u)=p_{j}(v)\\ 0 & otherwise \end{array}\right.$$

*S*_*u*,*v*_(*p*_*i*_, *p*_*j*_) = ∑_*i*,*j*_*T*_*u*,*v*_(*p*_*i*_, *p*_*j*_), where u and v are consecutive QSTs in x. *S*_*u*,*v*_ is the sum of all similarities between clustering method (clustering solution) *p*_*i*_ and clustering method *p*_*j*_. A consensus is reached between QST *u* and QST *v* when their corresponding vectors satisfy *S*_*u*,*v*_(*p*_*i*_, *p*_*j*_)/*m*>0.5, that is, when a majority of clustering models agree, as shown in Step 1. This finding indicates that more than half of the clustering methods assign *u* and *v* QSTs to the same cluster, as shown in Step 2. The CC ensemble algorithm is illustrated in Algorithm 2.

**Algorithm 2: Consensus Clustering Ensemble Method**

**Input:** Set of clustering solutions *p* = {*p*_1_,*p*_2_,….,*p*_*m*_}

**Output: Final** clustering *P*

BEGIN

Step i: find agreement *α* btween **clustering solutions *p* = {*p***_**1**_**,*p***_**2**_**,….,*p***_***m***_**} for each QST pair**

    For all QST pairs (*d*_*i*_, *d*_*j*_)

    *Begin*

    *v*_*i*_←{*p*_1_(*d*_*i*_),*p*_2_(*d*_*i*_),….,*p*_*m*_(*d*_*i*_)}

    *v*_*j*_←{*p*_1_(*d*_*j*_),*p*_2_(*d*_*j*_),….,*p*_*m*_(*d*_*j*_)}

            For each clustering solution *p*_*z*_ do

          If (*p*_*z*_(*d*_*i*_) = = *p*_*z*_(*d*_*j*_))

        *α* = *α*+1;

    *End if*

End for

If ($\frac{\alpha }{m}>0.5$)// m is the number of clustering solutions

    ***Step ii*: *if more than half of the methods uses d***_***i***_**, *d***_***j***_ ***in the same cluster*, *then create cluster C***_***u***_ ***and place them in it***

    Create *C*_*u*_ and *C*_*u*_←{*d*_*i*_, *d*_*j*_}

    *Else*

    ***Step iii*: *else if more than half of the methods uses d***_***i***_**, *dj in different clusters*, *then create clusters C***_***u***_ ***and C***_***a***_ ***and place each in different clusters***

    *C*_*a*_←*d*_*i*_

    Create *C*_*b*_ and *C*_*b*_←{*d*_*j*_}

    *End if*

    *End for*

End

## 4. Experimental results

### 4.1 Dataset

Clustering search engines use WSRC methods that integrate documents and provide descriptions for the collected documents. Thus, users find the required results effectively. The experiments are devised to use freely available text datasets and compare baseline models with recent clustering techniques. The datasets utilized for validation have been extensively used in several previous studies to validate the clustering techniques [29–31]. A range of gold standards has been used for investigating the search result clustering techniques, among which MORESQUE and ODP-239 are very popular. In the ODP-239 [28] technique, the documents are represented by a heading and a web summary, and the subtopics are selected from the uppermost layer of DMOZ6. The subtopics follow a normal distribution in MORESQUE because these subtopics are described depending on the uncertainty of Wikipedia [32]. Therefore, most query-related responses are included in the subtopics, but not all queries are related to Wikipedia or are vague. For instance, although the results of the search query produce several “world cup sports,” the query containing the keywords “world cup” entered in Wikipedia cannot be regarded as vague. Table 1 presents an overview of two datasets.

**Table 1:** Gold standard datasets of WSRC.

Dataset	No. of Queries	No. of Subtopics Average/Minimum/Maximum	No. of Snippets
**ODP-239**	239	10/10/10	25580
**MORESQUE**	114	6.7/2/38	11402

### 4.2 Evaluation metrics

This study uses the standard information retrieval metrics precision, recall, and f-measure [56–59] to evaluate the proposed model. Precision intends to evaluate the cluster based on the number of correctly retrieved instances out of the total number of retrieved queries. Recall aims to evaluate the cluster based on the number of correctly retrieved instances out of the total number of correct instances in the dataset. Each cluster is treated as if it is the result of a query, and each class is the desired set of documents for a query. The precision, recall, and F-measure of the cluster are calculated as follows:

$${\mathrm{Precision}}_{ci}=\frac{\#\mathrm{of}\;\mathrm{correct}\;\mathrm{instance}\;\mathrm{of}\;\mathrm{cluster}\;\mathrm{ci}\;\mathrm{given}\;a\;\mathrm{query}}{\mathrm{total}\;\#\mathrm{of}\;\mathrm{cluster}\;\mathrm{ci}\;\mathrm{instances}}$$ (8)

$${recall}_{ci}=\frac{\#of\;correct\;instance\;of\;cluster\;ci\;given\;a\;query}{total\#of\;instances\;given\;a\;query}$$ (9)

$$Precision=\frac{\sum_{i=1}^{number\;of\;clusters}Precision(C_{i})}{number\;of\;clusters}$$ (10)

$$recall=\frac{\sum_{i=1}^{number\;of\;clusters}recall(C_{i})}{number\;of\;clusters}$$ (11)

Now, the f-measure can be calculated as follows:

$$f-measure=\frac{2\times Precision\times Recall}{Precision+Recall}$$ (12)

## 5. Results

This section presents the results of the multiview multirepresentation on two k-based clustering methods, namely, k-means and k-medoids for WSRC. Other important results are from the conducted experiments of the proposed MMCC compared with single-view clustering methods. The following subsections present and explain the results in detail.

### 5.1 Results of multiview multirepresentation with individual clustering methods on ODP-239 dataset

This subsection presents the results of three views and representations, namely, QSTNVR, QSTWVR, and QSTDVR with k-based clustering methods, namely, k-means and k-medoids, for WSRC with the ODP-239 dataset.

Tables 2–4 show the results in terms of precision, recall, and f-measure value using the ODP-239 dataset. Each clustering method behaves differently under QSTNVR, QSTWVR, and QSTDVR. The performance of k-means differs from one view to another. Similarly, the performance of k-medoids varies from one view to another, that is, the f-measure values that represent the quality of clustering differ for the three views.

**Table 2:** Performance precision of QSTNVR, QSTWVR, and QSTDVR with k-means and k-medoids on the ODP-239 dataset.

**Clustering Methods**	**QSTDVR**	**QSTWVR**	**QSTNVR**
**k-means**	84.16	84.13	71.73
**k-medoids**	81.3	79.85	75.46

**Table 3:** Performance recall precision of QSTNVR, QSTWVR, and QSTDVR with k-means and k-medoids on the ODP-239 dataset.

Clustering Methods	QSTDVR	QSTWVR	QSTNVR
**k-means**	83.12	84.20	71.07
**k-medoids**	78.03	82.45	71.05

**Table 4:** Performance (f -measure) of QSTNVR, QSTWVR, and QSTDVR with k-means and k-medoids on the ODP-239 dataset.

Clustering Methods	QSTDVR	QSTWVR	QSTNVR
**k-means**	84.12	84.36	72.7
**k-medoids**	82.3	81.45	75.3

Tables 2–4 also show that QSTWVR outperforms QSTNVR and QSTDVR for both clustering methods (k-means and k-medoids). This finding may be due to the ability of QSTWVR to learn dense and low-dimensional word embeddings, and efficiently capture the implicit semantics of words; thus, it is more appropriate for short text [44].

Most importantly, with QSTWVR and QSTDVR, the best results obtained are those of k-means (with f-measures of 84.36 and 84.12, respectively), and the best result for QSTNVR is that of k-medoids with an f-measure of 75.3.

### 5.2 Results of the three multiview multirepresentation with clustering methods on the MORESQUE dataset

This subsection shows the results of QSTNVR, QSTWVR, and QSTDVR with k-means and k-medoids, for WSRC, on the MORESQUE dataset. Tables 5–7 present the results in terms of precision, recall, and f-measure on the MORESQUE dataset.

**Table 5:** Performance (precision) of QSTNVR, QSTWVR, and QSTDVR with k-means and k-medoids on the MORESQUE dataset.

Clustering Methods	QSTDVR	QSTWVR	QSTNVR
**k-means**	84.66	84.76	75.46
**k-medoids**	81.16	83.65	77.07

**Table 6:** Performance (recall) of QSTNVR, QSTWVR, and QSTDVR with k-means and k-medoids on the MORESQUE dataset.

Clustering Methods	QSTDVR	QSTWVR	QSTNVR
**k-means**	87.13	86.23	74.28
**k-medoids**	84.06	84.98	78.22

**Table 7:** Performance (f-measure) of QSTNVR, QSTWVR, and QSTDVR with k-means and k-medoids on the MORESQUE dataset.

Clustering Methods	QSTDVR	QSTWVR	QSTNVR
**k-means**	86.01	85.73	75.26
**k-medoids**	83.06	84.69	77.32

This section shows that the results of the multiview multirepresentation with individual clustering methods on ODP-239 dataset, k-means, and k-medoids behave differently with QSTNVR, QSTWVR, and QSTDVR. The performance of k-means and k-medoids differs from one view to another. Thus, the f-measure values that represent the quality of clustering are different for the three views.

However, Table 7 shows that QSTWVR and QSTDVR perform equally well (with f-measures of 83.06 and 84.69, respectively), and have better results than QSTNVR (f-measure = 77.32). Most importantly, for QSTWVR and QSTDVR, the best results are with k-means (f-measure values of 86.01 and 85.73, respectively). For QSTNVR, the best result is with k-medoids (f-measure = 75.26). However, QSTNVR has the worst performance using both clustering methods.

The comparative results of the clustering methods on both datasets show that clustering performance varies from dataset to dataset. However, the results obtained on the ODP-239 dataset are higher than those on the MORESQUE dataset due to the size, number of clusters per query, and different natures of the MORESQUE dataset. Moreover, different results can be obtained given the non-normally distributed data.

### 5.3 Results of MMCC ensemble

This section shows the results of MMCC ensemble on the ODP-239 and MORESQUE datasets. The CC ensemble method is constructed by combining three views and representations. V1, V2, and V3 (as shown in Tables 8 and 9) are used to denote QSTNVR, QSTWVR, and QSTDVR, respectively, for simplicity.

1. QSTNVR **(V1)**
2. QSTWVR **(V2)**
3. QSTDVR **(V3)**

**Table 8:** Performance of proposed MMCC on the ODP-239 dataset.

	V3C1		V3C2	
	V2C1	V2C2	V2C1	V2C2
**V1C1**	89.33	86.2	90.25	89.62
**V1C2**	89.92	89.62	91.15	87.17

**Table 9:** Performance of proposed MMCC on the MORESQUE dataset.

	V3C1		V3C2	
	V2C1	V2C2	V2C1	V2C2
**V1C1**	88.04	85.26	89.68	88.67
**V1C2**	89.23	88.85	89.79	86.47

For example, the column name “V3C1” in Tables 8 and 9 refers to the QSTDVR as V3 and k-means as C1.

All possible combinations use k-means (C1) and k-medoids (C2) methods. CC ensemble models must use all three views and representations to create a set of different candidate solutions and select high-quality clusters from the candidate solutions and form a superior clustering solution.

Table 8 displays the f-measure results of the proposed MMCC models on the ODP-239 dataset. All MMCC models outperform all previous clustering methods presented in Tables 4 and 6 for WSRC on the ODP-239 dataset. Table 9 displays the f-measure results of the proposed MMCC models on the MORESQUE dataset. The results show that MMCC models outperform the previous clustering methods in Tables 4 and 7 for WSRC on the MORESQUE dataset.

The results in Tables 8 and 9 also show that all MMCC models overcome the instability problem of single-view clustering models, and MMCC clustering models are stable and suitable for all types of data. In this study, “stable” is a reference on a method/algorithm that can achieve the best results on all given datasets.

**Fig 4:**
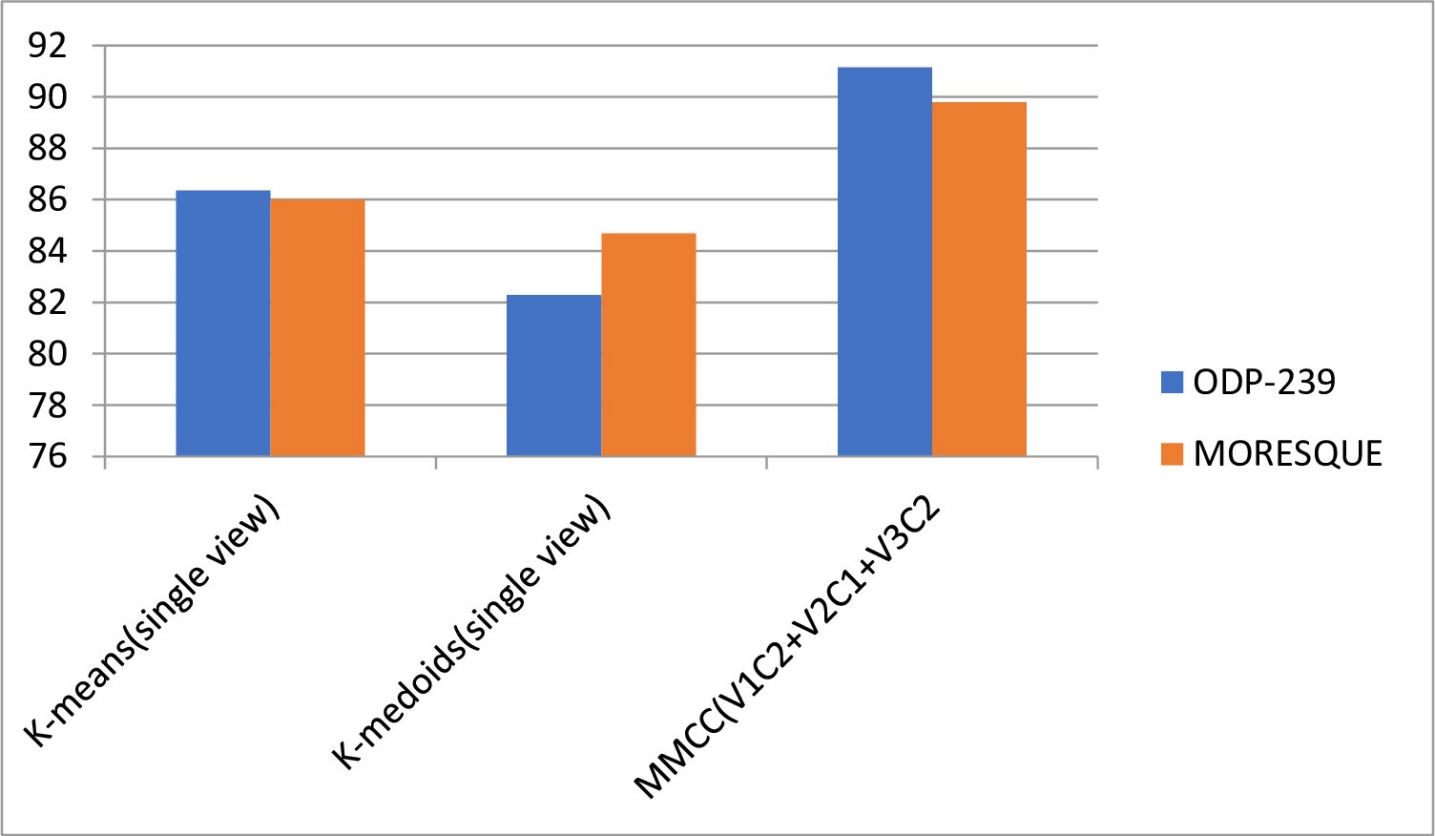
Performance comparison of the single view and MMCC.

Fig 4 shows that the results of MMCC models improve compared with those of all single-view clustering algorithms.

## 6. Discussion

This study investigates the performance of the overall multiview multirepresentation with conventional clustering technique, such as k-means and k-medoids, for WSRC and determines which algorithm provides better results. This study also presents the performance of proposed multiview multirepresentation clustering models that combine n-gram weighting, word embedding Word2vec, and DMM to generate different candidate clustering solutions. The incorporation of the proposed multiview multirepresentation based on consensus clustering ensembles (MMCC) enhances the performance of WSRC. The study aims to create a set of different candidate solutions and select high-quality clusters to form a superior clustering solution. Subsequently, the proposed MMCC improves the overall performance of WSRC compared with that of single-view clustering algorithms, as shown in Fig 4.

**Fig 5:**
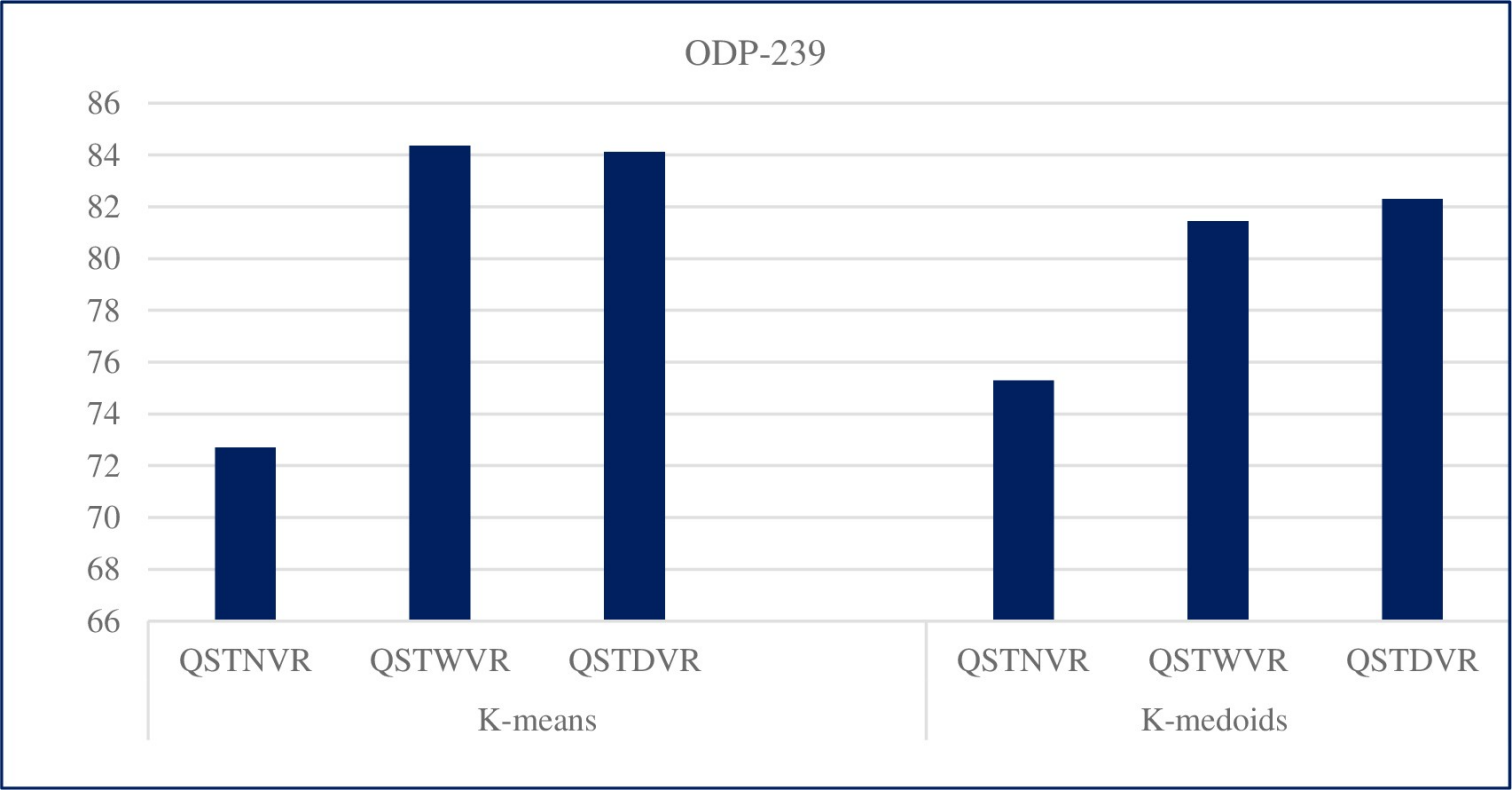
Performance of the three single-view models with k-means and k-medoids on the ODP-239 dataset.

This study investigates the effect of the proposed MMCC for WSRC on two standard datasets. Investigation is performed by comparing single-view clustering models with the MMCC model. Single-view clustering models, which use k-means and k-medoids, achieve good clustering performances. The performance of single-view clustering results obtained using the ODP-239 dataset with QSTNVR, QSTWVR, and QST-DVR with k-means are 72.7, 84.36, and 84.12, respectively. Similarly, the performance of single-view clustering results obtained using the ODP-239 dataset with QSTNVR, QSTWVR, and QST-DVR with k-medoids are 75.3, 81.45, and 82.3 respectively. Fig 5 displays the overall performance comparison of the three single-view models with k-means and k-medoids on the ODP-239 dataset.

By contrast, using k-means for the ODP-239 dataset shows a high result of 84.36 in terms of f-measure with QSTWVR. This finding may be due to the ability of QSTWVR low-dimensional word embeddings to capture the implicit semantics of words efficiently; thus, it is more appropriate for short text [44, 60]. Likewise, the ODP-239 dataset result obtained with k-medoids is 82.3 in terms of f-measure using QSTDVR, which is also high compared with other clustering methods.

**Fig 6:**
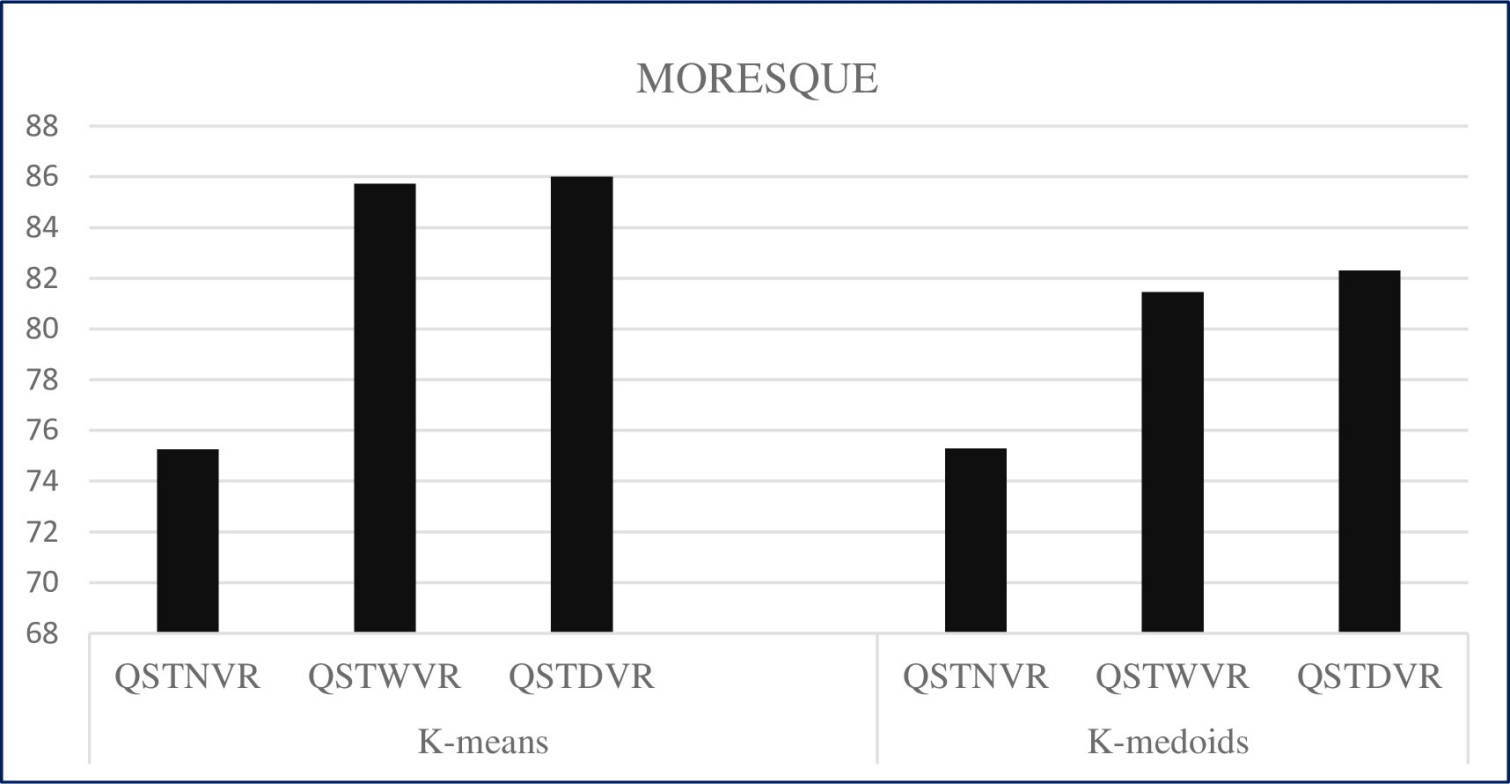
Performance of the three single-view models with k-means and k-medoids on the MORESQUE dataset.

The performances with single-view clustering results on the MORESQUE dataset using k-means based on three views, QSTNVR, QSTWVR, and QSTDVR, are 75.26, 85.73, and 86.01, respectively. Similarly, the performances of k-medoids with three views of QSTNVR, QSTWVR, and QSTDVR are 75.3, 81.45, and 82.3, respectively. Fig 6 displays the overall performance of the three single-view models with k-means and k-medoids on the MORESQUE dataset.

By contrast, using k-means obtains a high f-measure of 86.01 on the MORESQUE dataset using QSTDVR. The f-measure obtained using k-medoids is 82.03 using QSTDVR, which also achieves high results compared with other clustering methods. The comparative results of the clustering methods on both selected datasets demonstrate that clustering performance varies from dataset to dataset. However, the results on the ODP-239 dataset are higher than those on the MORESQUE dataset due to different results that can be obtained given the non-normally distributed data, size, number of clusters per query, and different natures of the MORESQUE dataset.

The stability of a clustering model can be defined as its ability to achieve the same results (performance) for all data types as shown in Figs 5 and 6. However, single-view clustering models are known for their instability, and no single clustering model is appropriate for all data types. The comparative results of the baseline clustering model on all datasets show that clustering performance varies from one dataset to another. The results in Tables 4 and 6 present the performance of each single-view model with k-means and k-medoids, and the presented results vary on different datasets.

In Subsection 5.3, the experimental results of the MMCC ensemble in Tables 8 and 9 demonstrate that a dynamic clustering model can be constructed by combining different candidate clustering solutions. These candidate clustering solutions combine high-quality and low-quality clusters to handle the dynamic nature of WSRC, where different queries indicate different search results and cluster numbers.

**Fig 7:**
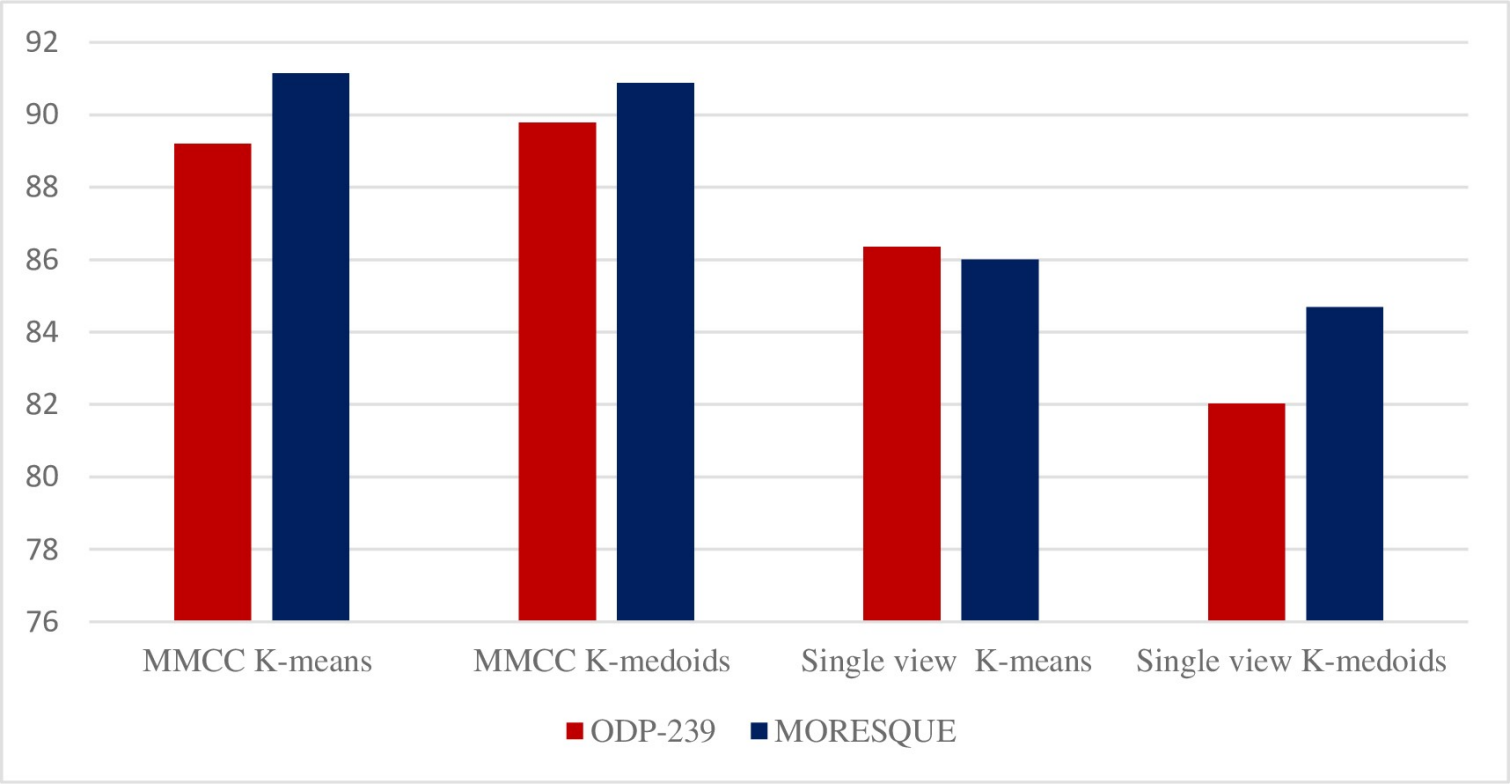
Overall performance of the single-view model and MMCC on the ODP-239 and MORESQUE datasets.

The experimental results in Tables 8 and 9 show that the proposed MMCC performs better than all single-view and representation clustering models. The performances of the single-view models with k-means and k-medoids are 86.36 and 82.03, respectively, on the ODP-239 dataset. Similarly, the performances of the single-view model with k-means and k-medoids are 86.01 and 84.69, respectively, on the MORESQUE dataset. However, the performances of the MMCC with k-means and k-medoids are 91.15 and 89.79, respectively, on the ODP-239 dataset. Integrating multiview methods with clustering methods is useful and improves the performance for all targeted datasets, as presented in Fig 7.

Applying multiple views leads to various clusters in different candidate clustering solutions. Using multiview multirepresentation simultaneously generates better clusters [27]. Hence, using multiple views that represent data in totally different representations with clustering methods and create a diverse set of candidate clustering solutions is logical. Multiview multirepresentation creates a diverse cluster and selects high-quality clusters from candidate clustering solutions, thereby leading to better clustering approach.

Additionally, the Wilcoxon rank statistical test [61] is used in this study to determine the significance of the results obtained by the MMCC model compared with the single-view models. A significance level *α* of 0.05 is employed for the test. Overall, the results show that the MMCC is statistically more significant than the other compared clustering methods on all datasets with a p-value of 0.014.

In summary, this study enhances WSRC using the proposed MMCC that combines different views and representation methods to create different candidate clustering solutions. The proposed MMCC improves the performance of WSRC by achieving better results. Similarly, this study investigates all clustering methods’ performance by conducting a statistical analysis, and the results show that the proposed MMCC is statistically significant over the compared methods.

## 7. Conclusion

This study presents a multiview multirepresentation approach with a CC ensemble method, which is also known as MMCC. This method uses multiple views to create a set of candidate solutions and selects high-quality clusters from the candidate solutions to form a superior clustering solution. This study contributes to improving the WRSC results by using three views and representations, namely, QSTNVR, QSTWVR, and QSTDVR with k-based clustering methods, namely, k-means and k-medoids, to generate the ensemble. These views and representations are used to create an initial set of candidate clustering solutions. This approach creates a diverse candidate clustering solution with combined high-quality and low-quality clusters. This study also proposes a radius-based cluster number estimation algorithm to handle the dynamic nature of WSRC. Two benchmark datasets are used to evaluate the proposed method and investigate the clustering techniques, among which MORESQUE and ODP-239 are extensively used. Moreover, the proposed MMCC outperforms the other methods in terms of precision, recall, and f-measure. The proposed method MMCC has a statically significant enhancement compared with other clustering methods based on the single-view model. Therefore, producing various sets of clusters from meaningful multiple views plays an essential role in WSRC clustering, but the existing clustering methods, including the proposed method, are vulnerable to poor cluster labeling and poor clustering quality due to overlapping problems. For future work, a new clustering method to address these limitations is proposed, and cost effectiveness and computational complexity can be considered. The current clustering methods attempt to cope with the drawbacks of the text data but cannot utilize the data with different representation methods to enhance clustering methods. Hence, better results in clustering methods could be achieved by employing data representation. On this basis, future researchers are encouraged to exploit features in enhancing text clustering methods from different views.
